# The Role of Calmodulin Binding Transcription Activator in Plants under Different Stressors: Physiological, Biochemical, Molecular Mechanisms of *Camellia sinensis* and Its Current Progress of CAMTAs

**DOI:** 10.3390/bioengineering9120759

**Published:** 2022-12-02

**Authors:** Shah Zaman, Syed Shams ul Hassan, Zhaotang Ding

**Affiliations:** 1 Tea Research Institute, Shandong Academy of Agricultural Sciences, Jinan 250100, China; 2 Shanghai Key Laboratory for Molecular Engineering of Chiral Drugs, School of Pharmacy, Shanghai Jiao Tong University, Shanghai 200240, China; 3 Department of Natural Product Chemistry, School of Pharmacy, Shanghai Jiao Tong University, Shanghai 200240, China; 4 Tea Research Institute, Qingdao Agricultural University, Qingdao 266109, China

**Keywords:** abiotic stressors, Ca^2+^ ion, CAMTAs, *C. sinensis*

## Abstract

Low temperatures have a negative effect on plant development. Plants that are exposed to cold temperatures undergo a cascade of physiological, biochemical, and molecular changes that activate several genes, transcription factors, and regulatory pathways. In this review, the physiological, biochemical, and molecular mechanisms of *Camellia sinensis* have been discussed. Calmodulin binding transcription activator (CAMTAs) by molecular means including transcription is one of the novel genes for plants’ adaptation to different abiotic stresses, including low temperatures. Therefore, the role of CAMTAs in different plants has been discussed. The number of CAMTAs genes discussed here are playing a significant role in plants’ adaptation to abiotic stress. The illustrated diagrams representing the mode of action of calcium (Ca^2+^) with CAMTAs have also been discussed. In short, Ca^2+^ channels or Ca^2+^ pumps trigger and induce the Ca^2+^ signatures in plant cells during abiotic stressors, including low temperatures. Ca^2+^ signatures act with CAMTAs in plant cells and are ultimately decoded by Ca^2+^sensors. To the best of our knowledge, this is the first review reporting CAMAT’s current progress and potential role in *C. sinensis,* and this study opens a new road for researchers adapting tea plants to abiotic stress.

## 1. Background and Importance of *Camellia sinensis*

**Figure 1:**
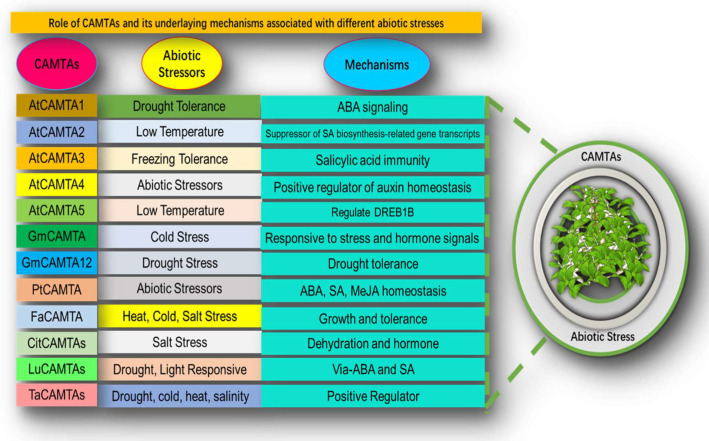
The role of CAMTAs and their underlying mechanisms associated with different abiotic stresses.

Tea (*C. sinensis* Kuntze) is a kind of evergreen woody tree that is one of the earliest crops in the world, and before tea was ever used as a beverage, people in ancient East Asia consumed it as food for decades. In fact, most of the tea is found in Asian geographical locations, including, China, India, and Sri Lanka, and on the other side in Kenya; some Caucasian countries, such as Turkey, Georgia, Russia, and Azerbaijan, are also famous for tea production. Surprisingly, China, India, Kenya, and Sri Lanka are the top four tea-producing nations in the world, and their combined output accounts for 75% of estimated worldwide tea production. In 2020, among the whole world’s tea production rate, China produced 42%, whereas India contributed 20%. The worldwide tea production was 7.0 million tons in 2020. However, Kenya, Argentina, and Sri Lanka were secondary producers [1]. In general, tea plant production is most successful in soils with a pH range of 4.5–6.5, high moisture levels, and average temperatures. Green tea is produced mainly from the *C. sinensis* species through swift roasting of fresh leaves to prevent oxidation, and its popularity is growing not only for its pleasurable flavor but also for numerous health advantages for humans, such as anti-inflammatory, anti-cancer, anti-obesity, and anti-allergic effects. Green tea intake has also been associated with the prevention of several types of cancers, such as lung, colon, esophagus, mouth, stomach, small intestine, kidney, pancreas, and mammary glands. Clinical studies have shown that green (black and Oolong teas to a limited extent) may minimize the risk of numerous chronic diseases, with this beneficial impact associated with the presence of substantial concentrations of polyphenols, which are effective antioxidants. Green tea, notably, could lower blood pressure, thereby reducing the risk of coronary heart disease and stroke. Perhaps, the consumption of green tea might prevent the risk of coronary heart disease by decreasing blood glucose levels and weight gain. Due to numerous health advantages, the relationship between tea consumption, mainly green tea, and people’s health has increasingly been known [2]. As a result of an increase in consumer demand for tea, the amount of land dedicated to tea cultivation is expanding across tea-growing regions, including China. Because tea plants live for such a long time, their physiology must be able to adapt to varying climates to survive in the extreme environments, which they encounter every year [3]. One of the major research areas for this undertaking is molecular mechanisms that are implicated in response to environmental stimuli. Abiotic stressors trigger plant genes and transcription factors, including CAMTAs. This novel transcription activator is also called signal responsive (SR) protein or ethylene-induced CaM-binding proteins (EICBP), discovered by [4] in parsley. It has been discovered that CAMTA transcription factors play very important, effective roles in plant growth and development under abiotic stress, such as low temperature, hormones, high salt, and drought. The CAMTA3 gene plays a key role in cold resistance in *Brassica napus* and two genes, ZmCAMTA4 and ZmCAMTA6, were overexpressed in maize under abiotic treatment, and cis-element study proves the active participation of CAMTA genes are associated with environmental stress and Positive regulator of auxin homeostasis in *A. thaliana* [5]. CAMTA transcription factors were found in different organisms, such as *P. tetraurelia* and *T. thermophila* [6]. Additionally, the orthologs of CAMTAs were found in *A. thaliana* [7], tobacco [8], cotton [9], poplar [10], soybean [11], barrel clover [12], strawberry [13], corn [14], and grapevine [15]. The transcription factor CAMTA has been investigated in detail for its role in both the biology of stress and development. Recently Li et al. (2022), identified the expression analysis of the CAMTAs family genes discovered in the genome of the “ShuChaZao” tea plant cultivar and the author stated that CsCAMTA1/3/4/6 expression levels were significantly higher in the cold-resistant cultivar ‘LongJing43’ than in the cold-susceptible cultivar “DaMianBai” during the cold acclimation stage in tea. Therefore, morphological, physiological, biochemical, and molecular studies are needed on tea cultivars and an understanding of sophisticated functions of CAMTAs to cope with abiotic stresses for the enhancement of growth, development, and quality of plants (Figure 1).

## 2. *Camellia sinensis* under Low Temperatures Stressors

Tea production and quality are affected in a spatially and temporally variable manner by the factors that are driving climate change. Although there has been a significant amount of research conducted regarding the effects of different stressors on the yield of food crops, the effects of abiotic stresses specifically on cold stress and low temperature on tea have gotten much less attention. However, recently, the problem has received a considerable amount of attention, and under the jurisdiction of the Food and Agriculture Organization (FAO) of the United Nations (UN), multilateral steps have been taken to address the impacts of climate change on tea [3]. Every year, a specific or typically a combination of distinct stress conditions can result in a significant loss of tea output and degradation of tea quality owing to leaf senescence. The efforts to improve tea’s cold resilience have been intensifying. Tea plant cold resilience has potential genetic characteristics, and cold acclimation can stimulate the development of this potential and provide ultimate cold resistance. The significant cold stress damage discovered in China shows that cold fluctuations are more damaging to tea production than heat extremes in the present climate. According to a study by Chen et al. (2018) that examined data from 1972 to 2014, the tea crop in Fujian province, eastern China, was more vulnerable to cold stress than heat stress [16]. Cold stress is particularly extreme in regions above 25° N, with a greater yield loss of 56.3% recorded in Shandong province [17]. In fact, due to the regular occurrence of harsh winters, such as freezing and low temperatures, cold spells etc., the growth, development, yield, and quality have all been significantly hindered in recent years [18]. When a plant is exposed to cold, it undergoes several physiological and biochemical changes as a result of molecular regulation, which uses a diverse range of signal transduction (ST) and transcription factors (TF) to reconfigure the expression of genes to modify the metabolism and development of plants under cold stress [19].

## 3. Physiological Mechanisms Associated with *Camellia sinensis*

The process through which plants react to stress is dynamic and sophisticated. As a result, its physiological and biochemical reactions can be used as a starting point in the search for and subsequent selection of resistant individual varieties and germplasm. Stomatal closure, inhibition of cell proliferation and photosynthesis, the buildup of organic osmolytes, and stimulation of respiration are all examples of such responses [20]. Plant systems, including physiological indicators, disrupt under different abiotic stressors. Photosynthesis, oxygen, and water retention are the main indicators and are impacted by climate change-induced stressors. Reduced carbon dioxide (CO_2_) concentrations transport to the chloroplast causes metabolic restrictions in tea plants that display the C_3_ mode of photosynthesis, a crucial activity influenced by water deficiencies [21]. The severity and frequency of stresses determine the relative impact of such conditions. Tea plants suffer damage to their photosynthetic machinery when they are subjected to stressful conditions, which in turn reduces the stomatal conductance of the leaves and turns their net photosynthesis and respiration rates. Scientific studies among both resistant and susceptible when subject to different stressors tea cultivars have found that the rate at which photosynthesis and respiration occur increases and decreases. Respectively, soil moisture also decreased [22,23]. With respect to air potential and saturation deficiencies, tea has a crucial xylem water potential value of 0.7 to 0.8 megapascals (MPa). Relative water content (RWC) is one of the most essential markers of plant water status when plants are exposed to abiotic stressors [24]; RWC varies by genotype, with resistant genotypes sustaining higher (RWC) than susceptible genotypes [23]. Different abiotic stressors, such as salt, cold, and drought and low and high temperatures, including chilling and freezing stresses, alter the tea properties on morphological, physiological, biochemical, and molecular levels [25].

## 4. Biochemical Mechanisms Associated with *Camellia sinensis*

Biochemical attributes in tea play a fundamental role under abiotic stressors, however, the influence of solute concentration on osmotic potential (OP) decreases when water is depleted from the cells and if solutes are constantly generated during cellular water loss, the (OP) will decrease faster than would be expected from the concentration effect solely. These include the accumulation of different metabolites, such as sugars and sugar alcohols, as well as amino acids, such as proline (Pro) and quaternary and other organic compounds such as glycine betaine and polyamines, (e.g., mannitol, trehalose, and galactinol). As a result of its importance in maintaining cellular integrity, scavenging free radicals, and stabilizing redox potential under stress [26]. It has been demonstrated that the level of (Pro) accumulation under stress in tea is highly connected with the plant’s ability to withstand stress, and it has also been revealed that the level of (Pro) concentration in stress-tolerant plants is higher than in stress-sensitive plants. On the other hand, tea plant biochemical properties are affected by abiotic stresses including cold/low temperatures resulting in novel biological behaviors of the cultivar. In fact, fatty acid biosynthesis is generally present in plant plastidial compartments in the form of membrane permeability. However, cold or low temperature restricts the growth and physiological attributes and damage the membrane fluidity, and creates membrane rigidification due to the creation of ice accumulation in the cellular compartments of tea plants, but fatty acid desaturase maintains the membrane permeability and increase stress tolerance under abiotic stressors including cold/low temperatures. Zaman et al. (2019) reported that concentrations of fatty acid altered with phenotypic diversity, and fatty acid desaturase stimulates the formation of alpha-linolenic acid from linoleic acid in tea under abiotic stresses, including mild and extremely cold conditions [24]. Biochemical analysis of amines, amino acids, carotenoids, organic acids, phenolic acids, sugar alcohols, and sugars are useful indicators for tea and these metabolites promote the evolution of cultivars that are more resistant to abiotic stressors, such as cold/low temperature.

## 5. Molecular Mechanisms Associated with *Camellia sinensis*

**Figure 2:**
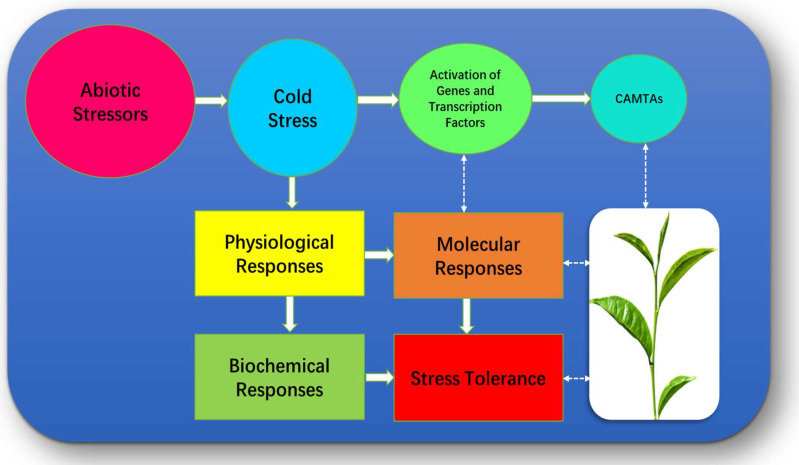
Factors associated with *C. sinensis* under Cold temperature.

Some genotypes can cope with different abiotic stressors and sustain their tolerance mechanisms. For example, differentiating between cold/drought tolerant and drought/cold susceptible tea cultivars based on their molecular profile could help to boost crop yields in response to combined Stress [27]. As a result, a rising number of scientists are concentrating their studies on how to develop the stress resistance of tea plants. Several reports have documented how stress impacts essential tea properties and their synthetic gene precursors on physiological, biochemical, and molecular levels, which are involved in tea reaction and tolerance to stress [20,23], including cold stress [25] (Figure 2). These factors can be utilized as a stress indicator for screening and clonal selection; therefore, they will provide theoretical knowledge to produce climate-resilient tea cultivars under different stressors. The major research areas for this undertaking are molecular mechanisms that are implicated in response to environmental stimuli. During abiotic stressor plants activate numerous genes and transcriptional factors. CAMTAs are one of them. CAMTAs also known as (SR) protein or (EICBP), is thought to be the most comprehensive defined family of (CBPs). The involvement of this unique protein which interacts with DNA was discovered for the very first time during the process of isolating a partial cDNA clone (CG-1) from *P. crispum* by [4]. Later, Yang and Poovaiah (2000) discovered that NtER1 is a (CBPs) that is involved in ethylene-regulated plant death and senescence, which indicates that it may have a potential application in the increase of the storage life of horticulture crops. In addition to this, they demonstrated that CBPs to NtER1 in a Ca2C-dependent manner. They also reported that specified probable implications of CAMTAs were regulated in several signal transduction pathways in plants [28]. CAMTA has since been found in a variety of multicellular organisms. Proteins identical to CAMTA have been found in unicellular eukaryotes such as ciliates, *P.tetraurelia*, and *T.thermophila* [6]. Additionally, the orthologs of CAMTAs were found in *A. thaliana* [7], tobacco [8], cotton [9], poplar [10], soybean [11], barrel clover [12], strawberry [13], corn [14], and grapevine [15]. The CAMTA has been investigated in detail for its role in both the biology of stress and development. Several researchers have examined the role of CAMTA genes response under biotic stress conditions as well as CAMTAs transcriptional response under abiotic stress conditions, which includes cold stress [5,29,30]. Remarkably, the decoding of the CAMTA gene expression patterns and functional redundancy in model plants and a variety of crops established the groundwork for additional research into the role of these novel genes under abiotic stresses, which has the potential to be beneficially applied to the agricultural sector. However, a comprehensive review of gene precursors or signaling on the molecular level on tea plants under abiotic stressors including cold/low temperature is still missing. Taken together, some important studies have shown that Ca^2+^ and CAMTAs are important in coping with various stresses in different plants, and several genes involved in Ca^2+^ (e.g., CsCBLs, CsCDPKs, CsCIPKs, and CsCMLs) were up-regulated under stress conditions [31]. On the other hand, despite being the major (CBPs) in the calcium signaling pathway, the functions of CAMTAs in tea plants have not been studied considerably. The present review summarizes the advancement made in the recent years of CAMTAs role in different plants adoptions to abiotic stressors as well as a current progress of CAMTAs role in *C. sinensis*.

## 6. Role of Calmodulin Binding Transcription Activator in Plants

**Figure 3:**
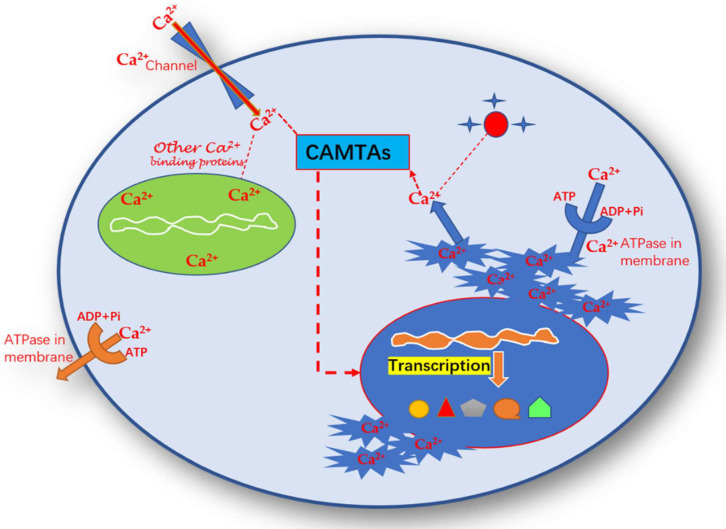
Mode of action of calcium signaling of Ca^2+^ with CAMTAs for the regulation of transcriptional factors for triggering the genes and transcription factors.

CAMTAs, has an evolutionarily conserved structure and is found in a wide variety of eukaryotes. It promotes plant growth and development and plays a role in how plants react to environmental stresses. Ca^2+^ is a ubiquitous secondary messenger that acts as a primary sensor and regulator of plants in dealing with growth, development, and varied environmental stressors [31]. Until now, CBPs also known as CAMTAs, and (SR) proteins, or ethylene (ET) induced (CBPs) have been shown to mediate the whole life cycles of multicellular eukaryotes, including plants and humans [6]. In plants, it is obvious that CAMTAs have 6 unique components, namely nuclear localization signals, which participate in targeting the protein in the nucleus, and the CG-1 domain [28]. Several CAMTAs genes have been identified from many plant species, including 6 AtCAMTAs in *A. thaliana*, 10 VvCAMTAs in grape [15], 9 ZmCAMTAs in maize [14], 7 SlCAMTAs in tomato [18], 7 MsCAMTAs in alfalfa [32], 9 LuCAMTAs in flax [33], and 9 CsCAMTAs from citrus [34]. CAMTAs have been demonstrated to play significant roles in the maintenance of plant growth and development with different hormones under biotic and abiotic stress responses including low-temperature stress responses [35,36]. Extensive biochemical and genetic investigations have thoroughly clarified the role of Ca^2+^ as a major nutrient in supporting the structural integrity of cell walls and influencing numerous physiological processes under biotic and abiotic stressors [7,37]. Ca^2+^ spikes are typically the result of diametrically opposed events in cells: Ca^2+^ influx (entry) through dedicated channels or Ca^2+^ efflux (exit) through distinct pumps [38]. In fact, under a cold environment, elevated Ca^2+^ levels could stimulate the interaction of CAMTAs with the cis-acting element, and then trigger the expression of numerous signaling transcriptions. So, The Ca^2+^/CaM complex modulates interactions between sensory stimuli and transcription, (Figure 3), allowing plants to react immediately to cold exposure and to improve their cold adaptation and resistance [39]. CAMTAs itself is a group of transcription factors (TFs) that adjust plants when subject to cold or low stress in a Ca^2+^dependent manner. There is a significant amount of evidence that suggests Ca^2+^ mediated signaling and tune CAMTAs, which are involved in the transmission of stress cues, such as light [40,41], temperature [42], salt [43], cold [44], oxidative signals [45], reactive oxygen species (ROS) [46], ET as a hormone signals [47], abscisic acid (ABA) [48], gibberellins (GA) [49], and auxins (IAA) [50], play a crucial part in plants under cold/low temperatures (Table 1). According to Galon et al. (2010) AtCAMTA1-3 were negative regulators of auxin that linked to red light and high light responses, meanwhile AtCAMTA4-6 were considered to as positive integrators of auxin signaling and homeostasis [51]. Further study found that CAMTA1-3 may synergistically produce the maximum expressions of CBF1-3 after 2 h of 4 °C chilling treatment resulting in the up-regulation of more than 15% cold-regulated genes in the CBF independent mechanism and so strengthening *A. thaliana* freezing resistance [52]. Likewise, multiple stress-related cis-acting elements were found in the promoter sequences of various ZmCAMTA genes indicating that ZmCAMTAs play an important role in stress responses. ZmCAMTAs transcripts were significantly induced by maize rough dwarf disease infection with ZmCAMTA6/7a, exhibiting differential gene expression among disease-tolerant and disease-sensitive cultivars [14]. Apart from the AtCAMTAs, the functions of several CAMTAs in plants have been well investigated. Various stress and hormone treatments modulated the expression of eight CitCAMTAs genes in citrus [34]. The aforementioned information revealed the number of CAMTAs played a significant role in the maintenance of plant growth and development at environmental stimuli.

## 7. Role of Calmodulin Binding Transcription Activator under Different Stressors

**Figure 4:**
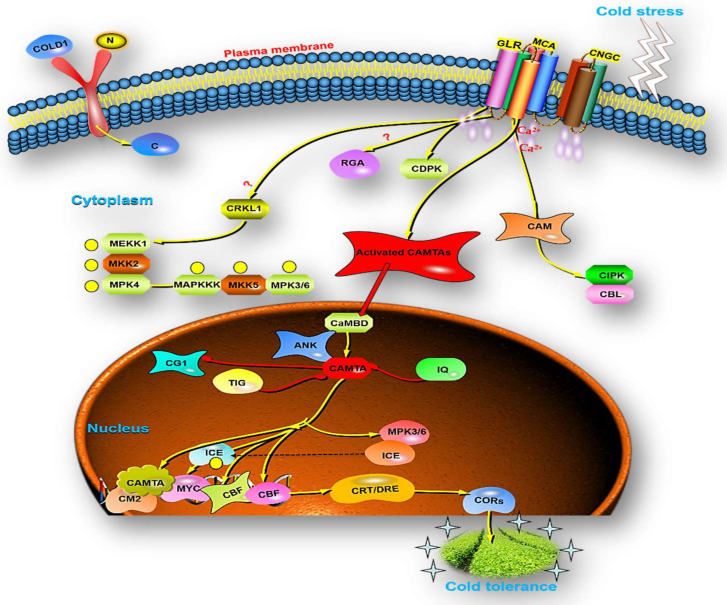
The systematic representation of Ca2+ and CAMTA interaction: CNGCs, GLRs, and MCA channels allow Ca2+ ions into the cytoplasm. Calmodulins (CaMs), CMLs, CBLs, and Ca2+-dependent protein kinases sense Ca2+ entering the plant cell (CDPKs). CRLK stimulates MEKK1–MKK2–MPK4 to positively regulate cold-triggered gene expression. CAMTAs induce CBF expression through the CM2 promoter motif and CBF protein activates the cold-resistant COR genes in plants to increase cold tolerance.

The processes that underlie the role of CAMTAs actions in response to abiotic stress have been unraveled to a reasonable degree [72]. Under typical circumstances in which plants are not subjected to any kind of stress, there is only a slight increase in the activity of the CAMTA genes. This could be caused by the fact that the genes in this family of TFs exhibit functional redundancy or it could be because the genes in this group are induced by certain environmental factors that are very distinct. Noteworthy, 6 CAMTAs were found in the model plant *A. thaliana*. Such as; CAMTA1, CAMTA2, CAMTA3, CAMTA4, CAMTA5, and CAMTA6. It was demonstrated that changes in the environment such as high temperatures, salt, hydrogen peroxide, and physical wounds, hormones such as ET, ABA, MeJA, and SA, all worked together and quickly trigger the regulation of CAMTAs genes [52]. In fact, AtCAMTA1, the very first member of the CAMTAs superfamily found in *A. thaliana*, a role for this gene has been documented in auxin signaling (increased expression of AUX/IAA-IAA29), transport, and its maintenance. The Beta-glucuronidase (GUS) plasmid exhibited cell-specific expression patterns of auxin when applied to agrobacterium tumefaciens that possessed the AtCAMTA1 promoter. The chemical inhibition of polar auxin transport provides evidence for the participation of CAMTA1 in the auxin signaling pathway. The vulnerability of CAMTA1 in auxin pathways is further supported by profiling of gene expression of the auxin transport inhibitor 1-N-naphthylphthalamic acid (NPA) in plants. A genome-wide analysis of the *camta1* mutant’s transcriptome discovered 63 genes with an up-regulation. Further, in an extensive study, the author found 17 genes were implicated in the (IAA) signaling pathway. In addition, the *camta1* mutants were much more sensitive to the impact of IAA on hypocotyl growth as contrasted to the wild type [51]. The researchers also found that CAMTA1, CAMTA2, and CAMTA3 are negatively responsible for the regulation of IAA, and they are aligned with genes that are involved for red light and high light responses. On the contrary side, CAMTA4, CAMTA5, and CAMTA6 are positive regulators of IAA, and they are attributed to the genes that are responsible for blue light and darkness responses [51]. In a comparable pattern, phytohormonal modulation of CAMTAs is also generalized to brassinosteroid (BR) signaling in which the protein BZR1, which is engaged in the BR signaling pathways has CAMTA5 as a binding protein [73]. More esoteric findings have revealed that when plants are infected with biotrophic or hemibiotrophic pathogens and grown under low temperatures (4 °C) for more than a week, the inhibition of genes associated with SA biosynthesis by CAMTA at high temperatures (22 °C) is overcome. In normal operating conditions CAMTA3 suppresses the salicylic route via an N-terminal repression module. The N-terminal repression module functions independently of CaM binding to the calcium-dependent CaM binding domain (CaMBD) or the calmodulin-binding motif IQ (IQXXXRGXXXR) motif domain. This observation is unique since the CAMTA3 repression statement includes the adherence of CaM to CaMBD. To summarize, CAMTA3 suppression activity at low temperatures and pathogen infection are governed by similar processes [74]. To clarify, three fundamental regulators of cold-responsive specific genes are dehydration-responsive element binding protein 1/C-repeat binding factors. Dehydration-responsive element binding (DREB1) triggers cold-responsive regulatory cascades and stimulates numerous genes under cold stress response. Plants recognize swift and sustained temperature drops as cold stress, which trigger the activation of DREB1 genes. CAMTA3 and CAMTA5 react quickly when the temperature drops suddenly by triggering the expression of DREB1s. However, CAMTA3 and CAMTA5 may not respond to gradual temperature decreases. Despite circadian clock attributed 1 and late prolonged hypocotyl (which operate as transcriptional regulators to regulate DREB1 expression solely throughout the day), AtCAMTA3 and AtCAMTA5 activate both during the day and at night [75]. In fact, CAMTA has five primary domains: CG-DNA binding motif, TAD-transcriptional activation domain, TIG-non-specific DNA interaction, Ankyrin repeats-protein–protein interaction, and CAMBD-CaM binding. Generally, cold stress opens Ca^2+^ channels in plants, allowing Ca^2+^ to enter cells quickly and transfer through Ca^2+^ sensor—CaM—senses, which increases the ([Ca^2+^] cyt). CaM regulates CAMTA gene as a transcription in a Ca^2+^ in a dependent way and inhibits the plant from responding to cold stress (Figure 4). Kakar et al. (2018) also found that CAMTAs enhanced the tolerance mechanism of *N. tabacum* under different stresses [76]. The above findings revealed that most of CAMTAs play an important role in different plant adaptation to biotic/abiotic stress including cold/low temperature (Table 2).

## 8. Current Progress of CAMTAs in *Camellia sinensis*

**Figure 5:**
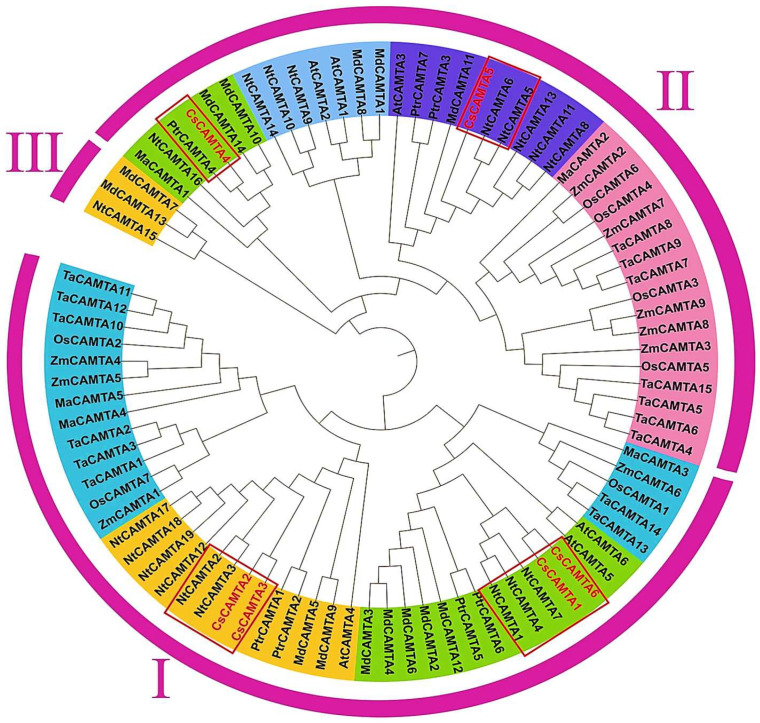
Phylogenetic analysis of CsCAMTAs and known CAMTAs of other plant species. The information and figure of CAMTAs phylogenetic tree were taken from the study of [18].

To the best of our knowledge, only the below mentioned studies were found on CAMTAs in *C. sinensis*. Recently, from the laboratory of well famous scientist of tea in northern China Professor Zhaotang Ding and his co-author Li et al. (2022), identified the expression analysis of the CAMTAs family genes discovered in the genome of the “ShuChaZao” tea plant cultivar; the author found that 6 highly conserved operational motifs were found in all CsCAMTAs, in which the author revealed in phylogenetic analysis all 88 CAMTAs were grouped into 3 subfamilies, including 4 CsCAMTAs in subfamily I (Figure 5). These identified CsCAMTAs were important in controlling tea plant aging and flowering times. Most CsCAMTAs were induced at distinct time points under hormone and abiotic stress conditions. Interestingly, CsCAMTA1/3/4/6 expression levels were significantly higher in the cold-resistant cultivar ‘LongJing43’ than in the cold-susceptible cultivar “DaMianBai” during the cold acclimation stage, however, CsCAMTA2/5 expression levels were greater in “DaMianBai” during the entire cold acclimation period [18].

Another recent study on the identification and expression analysis of CAMTAs genes in the genome of the “Quntizhong” tea plant reveal their complex regulatory role in stress responses in which the author reported 15 CAMTAs in group I and 9 CAMTAs in groups II and III in phylogenetic analysis. However, five CsCAMTAs genes were identified from the tea plant genome, which is considered to play an important role under different time points when exposed to stress [83].

However, Wang et al. (2013) found that 13 genes were found to be involved in signal transduction in the tea cultivar “Longjing 43” response to low-temperature stress; these genes were characterized as CDPKs, CBL, CAMTA, MAPK, and phospholipase. Nine of these genes were found to be slightly higher compared to CA1 (four calmodulin genes, two CDPK genes, one CAMTA gene, and two phospholipase genes), however, four were found to be down-regulated under low temperatures [35]. Taken all together, in this review we highlighted the role and current progress of CAMTAs in various plants under different stressors, including on *C. sinensis.*

## 9. Conclusions and Future Perspective

There has been a plethora of studies on physiological, biochemical, and molecular studies on the regulation of genes and transcription factors involved in cold signaling in plants. Cold stress activates a cascade of Ca^2+^ through various Ca^2+^ channels and/or Ca^2+^ pumps, which are then decoded by a panel of Ca^2+^ sensors to control the expression of genes and transcription factors associated with cold tolerance under low temperatures. Significant progress has been made in understanding the fundamental components of Ca^2+^ signaling and CAMTAs. No wonder, the interaction of Ca^2+^ signaling and CAMTAs are beneficial for the cold tolerance mechanism in different plants when they are subjected to cold or low temperatures. However, to the best of our knowledge CAMTAs in tea plants has received a limited amount of research compared to other plants. There is much less information available on Ca^2+^signaling and CAMTAs mechanisms associated with tea plants. Therefore, more research on functional genomics and cutting-edge technologies is essential to understand the molecular mechanisms of the CAMTAs gene family and transduction of calcium Ca^2+^ signaling in different cultivars of *C. sinensis* under abiotic stresses, including low temperatures.

## Figures and Tables

**Table 1:** Calcium signaling components in plants under different stressors.

Gene	Ca^2+^ Component	Species	References
*CAMTA5*	Transcription factor	*A. thaliana*	[53]
*MdCPK1a*	Ca^2+^sensor	*M. domestica*	[54]
*ZmCPK1*	Ca^2+^sensor	*Z. mays*	[55]
*VaCPK30*	Ca^2+^sensor	*V. amurensis*	[56]
*MaCDPK7*	Ca^2+^sensor	*M. acuminata*	[57]
*OsCPK24*	Ca^2+^sensor	*O. sativa*	[58]
*MeCIPK7*	Ca^2+^sensor	*M. esculenta*	[59]
*MsCML46*	Ca^2+^sensor	*M. sativa*	[32]
*CML21v1,*	Ca^2+^sensor	*V. amurensis*	[60]
*SlCML37*	Ca^2+^sensor	*S. lycopersicum*	[61]
*GhCAX3*	Ca^2+^ channel	*G. hirsutum*	[62]
*ANNEXIN1*	Ca^2+^ channel	*A. thaliana*	[63]
*GLR3.5*	Ca^2+^ channel	*L. lycopersicum*	[64]
*AtGLR1.3*	Ca^2+^ channel	*A. thaliana*	[65]
*AtCNGC4*	Ca^2+^ channel	*A. thaliana*	[66]
*ZjCNGC2*	Ca^2+^ channel	*Z. jujuba*	[67]
*CNGC9*	Ca^2+^ channel	*O. sativa*	[68]
*AtCAX1*	Ca^2+^ channel	*A. thaliana*	[69]
*Ca2C/cation antiporter*	Ca^2+^ channel	*Saccharum spp.*	[70]
*AtGLR3.4*	Ca^2+^ channel	*A. thaliana*	[71]
*CAMTA3*	Transcription factor	*A. thaliana*	[29]

**Table 2:** Roles of CAMTAs in plants under different stressors.

Gene Names	Role	Species	Reference
*CAMTA*	Biotic/abiotic stress	*P. trichocarpa*	[10]
*TaCAMTAs*	Drought, cold, heat, and salinity	*T. aestivum*	[36]
*FaCAMTA*	Heat, cold, and salt stress	*F. ananassa*	[13]
*ZmCAMTA*	Biotic/abiotic stress tolerance	*Z. mays*	[14]
*VvCAMTA1*	Ca^2+^ signal transduction	*V. vinifera*	[15]
*GmCAMTA*	Responsive to stress and hormone signals	*G. max*	[11]
*LuCAMTAs*	ABA, SA, drought, low temperature and light responsive	*L. usitatissimum*	[39]
*NtabCAMTAs*	Drought, cold, cadmium, and black shank stress	*N. tabacum*	[76]
*TaCAMTA4*	Negative regulator of defense response against P. triticina	*T. aestivum*	[77]
*BnCAMTA*	Stress-inducible and phytohormonal regulation	*B. napus*	[78]
*AtCAMTA6*	Na+ homeostasis in seed germination	*A. thaliana*	[72]
*AtCAMTA5*	BZR1-associated protein; BR signaling	*N. benthamiana*	[35]
*AtCAMTA4*	Positive regulator of auxin homeostasis	*A. thaliana*	[51]
*GmCAMTA12*		*Drought tolerance*	[79]
*AtCAMTA1*	Drought tolerance via ABA signaling	*A. thaliana*	[30]
*AtCAMTA2*	Suppressor of SA biosynthesis-related gene transcripts	*A. thaliana*	[80]
*AtCAMTA3*	Plant defenses against insect herbivory via SA-JA crosstalk	*A. thaliana*	[81]
*AtCAMTA5*	BZR1-associated protein; BR signaling	*N. benthamiana*	[73]
*CAMTA1*	Cold acclimatization	*A. thaliana*	[29]
*CAMTA2*	Activator of AtALMT1 (metal toxicity)	*A. thaliana*	[82]

## Data Availability

Not applicable.
